# The Spore Coat Protein CotE Facilitates Host Colonization by *Clostridium difficile*

**DOI:** 10.1093/infdis/jix488

**Published:** 2017-09-15

**Authors:** Huynh A Hong, William T Ferreira, Siamand Hosseini, Saba Anwar, Krisztina Hitri, Anthony J Wilkinson, Wilfried Vahjen, Jürgen Zentek, Mikhail Soloviev, Simon M Cutting

**Affiliations:** 1 School of Biological Sciences, Royal Holloway, University of London, Surrey; 2 Department of Chemistry, University of York, United Kingdom; 3 Institute for Animal Health, Freie University of Berlin, Germany

**Keywords:** *Clostridium difficile*, spores, colonization, chitinase, virulence, mucus

## Abstract

*Clostridium difficile* infection (CDI) is an important hospital-acquired infection resulting from the germination of spores in the intestine as a consequence of antibiotic-mediated dysbiosis of the gut microbiota. Key to this is CotE, a protein displayed on the spore surface and carrying 2 functional elements, an N-terminal peroxiredoxin and a C-terminal chitinase domain. Using isogenic mutants, we show in vitro and ex vivo that CotE enables binding of spores to mucus by direct interaction with mucin and contributes to its degradation. In animal models of CDI, we show that when CotE is absent, both colonization and virulence were markedly reduced. We demonstrate here that the attachment of spores to the intestine is essential in the development of CDI. Spores are usually regarded as biochemically dormant, but our findings demonstrate that rather than being simply agents of transmission and dissemination, spores directly contribute to the establishment and promotion of disease.

## Teaser

Spores of *Clostridium difficile* carry a chitinase enzyme whose function is to bind to mucin, leading to its degradation. We show in vivo that this is potentially an important element of colonization and virulence.

*Clostridium difficile* is a Gram-positive, spore-forming human and animal pathogen and one of the leading causes of nosocomial antibiotic-associated diarrhoea in developed countries [1]. The disease results from antibiotic-induced dysbiosis of the gut microflora that allows spores of *C. difficile* to germinate, proliferate, and produce at least 2 inflammatory cytotoxins (toxins A and B), resulting in tissue damage [2, 3]. It has been demonstrated that vegetative cell adherence is important and a number of adhesins have been found, including SlpA [4], flagellin [5], and Cwp84 [6]. However, whether it is necessary for the spore to attach to the intestine prior to germination is unknown.

During the acute stage of infection, there is transient production of enormous numbers of spores (approximately 6 logs higher than the infective dose) in the intestine [7]. However, the number of spores produced can vary significantly for different strains. A number of clues suggest that the spore may contribute to *C. difficile* infection (CDI). First, hamsters challenged with spores of a non-toxigenic strain of *C. difficile* (CD1342) experienced cell damage in the cecum as well as an inflammatory response [8]. Second, Kansau et al [9] found that in a mouse model, the sporulation process of hypervirulent strain R20291 was initiated earlier compared to non-hypervirulent strains. Finally, a *C. difficile spo0A* mutant that cannot produce spores was shown to be unable to persist within, and effectively transmit disease between mice [10].

Other than being an agent of transmission, an intriguing question that has not been addressed is whether the spore per se plays a role in the development of disease. The outermost layer of the spore carries a number of enzymes, but the CotE protein is particularly interesting [11]. It is an 81-kDa bifunctional protein that carries 2 distinct domains, an N-terminal peroxiredoxin domain and a C-terminal chitinase domain [11, 12]. The peroxiredoxin domain resembles a 1-Cys peroxiredoxin, suggesting it may be involved in reducing hydrogen peroxide arising as a by-product of SodA-mediated cross-linking of spore coat proteins [11]. The chitinase domain, belonging to the glycohydrolase family 18, might prima facie play a nutritional role in the turnover of macromolecules such as chitin. Intriguingly, CotE may have a more sophisticated function and contribute directly to pathogenesis.

In this article, we show in vitro that CotE enables spore adhesion to mucus via direct binding to the mucin glycoproteins GlcNAc and GalNAc. Our in vivo data demonstrate that spore attachment is important for developing CDI and that CotE is required for efficient intestinal colonization. Surprisingly, CotE increased the severity of CDI in a hamster model; animals dosed with spores lacking CotE exhibited significantly delayed symptoms of CDI. Taken together we suggest that CotE, and thus the spore, plays an integral role in attachment to the gut mucosa to initiate the infection and the ensuing virulence of CDI, a finding that is both important and hitherto unnoticed.

## METHODS

### Strains, Reagents, and General Methods

Two wild-type strains of *C. difficile* were used in this work: 630Δerm and 630. The 630Δerm strain is a spontaneously cured derivative of 630 (*tcdA*^*+*^*tcdB*^*+*^) that is erythromycin sensitive [13]; 630 (erythromycin resistant) was isolated from a patient with pseudomembranous colitis during an outbreak of CDI [14]. The 630 strain is used in animal experiments as the 630Δerm strain is sensitive to clindamycin. Two previously described [12] *cotE* ClosTron mutants, isogenic to 630Δerm, were used: ET46 (*cotE::CT220s*) and ET24 (*cotE::CT1203s*). A *sigK* ClosTron mutant JP051 (*sigK::CT266s*) carrying an insertion after codon 87 of the coding open reading frame was constructed in this laboratory and will be described elsewhere. The CotE mutants were complemented with wild-type copies of the respective genes using pRPF185 as described previously [12]. All spores used were purified through a 20%–50% Histodenz gradient. Expression of CotE on the spore surface was verified by Western blotting (Supplementary Figure 1*C*). Antibodies (mouse) recognizing the N-terminus of CotE (M1-K234) have been described previously [12] while antibodies to the C-terminus of CotE were raised in mice by 3 intraperitoneal injections of rCotEC (P381-F712) (Supplementary Figure 1). Methods for growth and sporulation of *C. difficile* and previously described methods are shown in the Supplementary Methods.

### Adhesion of Spores to HT29-MTX Cells

The method was performed as described previously [15]. In brief, cells were seeded at 4 × 10^4^ cells/well in 24-well plates for 14 days at 37°C with 7.5% carbon dioxide. On the day of experimentation, cells were washed once with phosphate-buffered saline (PBS). Spores were added to the wells and incubated for 2 hours at 37°C. Non-adherent spores were removed by washing with PBS 5-times and cells lysed with Triton X-100 (0.1% w/v) in PBS for 10 minutes before enumeration of adherent spores by plating on brain heart infusion broth supplemented with yeast extract, cysteine and sodium taurocholate (BHISS) plates. Percentage adhesion was calculated using the formula [% adhesion = (colony-forming units [CFU] count / initial number of spores added) × 100]. Data were presented as the percentage proportion of binding demonstrated by strain 630 spores. For antibody-blocking adhesion experiments; the same procedure as described above was used, but spores were preincubated with anti-CotEC antibody (1/10) for 30 minutes at 37°C before adding to the wells.

### Ligand Binding

High binding enzyme-linked immunosorbent assay (ELISA) plates were coated with ligands conjugated to human serum albumin (Dextra, Reading, United Kingdom) at a concentration of 10 µg/mL overnight at 4°C. For mucin, porcine stomach type III (M1778, Sigma), a solution was made fresh in PBS (pH 7.4) and used to coat plates at 1 µg/mL (overnight at 4°C). After blocking the plate with 2% (v/v) bovine serum albumin (BSA) in PBS (1 hour at 30°C), recombinant proteins (rCotEN or rCotEC) were diluted in 1% (v/v) BSA at a concentration of 0.5 μM and incubated for 2.5 hours at 30°C. rCotEN (mouse) and rCotEC (mouse) were used as primary antibodies and anti-mouse immunoglobulin G–horseradish peroxidase (DAKO P0447) was used as the secondary antibody.

### Mucin Degradation

Three milliliters of sterile molten 0.5% (w/v) agarose containing 0.5% (w/v) of mucin (porcine type III, Sigma M1778) was overlaid onto sterile microscope slides (76 × 26 mm) rapidly. The sterile slide was placed into a petri dish and the agarose left to solidify, after which 3-mm-diameter holes were punched out of the medium. Ten microliters of spores (1 × 10^10^/mL) was added to the wells. The petri dished was closed and sealed with parafilm and incubated for 48 hours at 37°C. Before staining with amido black solution for 30 minutes (Sigma A8181), the agarose was compressed to a thin layer using a 500-g weight (30 minutes with Whatman No. 3 between agarose and weight). The slide was then de-stained several times with 7% (v/v) acetic acid.

### Animal Experiments

All animal procedures were performed under the UK Home Office project license PPL 70/8276. For animal experiments, we followed methods from those described previously [15, 16].

#### Hamsters

Female Golden Syrian Hamsters were 16–18 weeks old (Harlan UK Ltd). For the hamster challenge, animals were dosed by intragastric gavage (i.g.) with clindamycin (clindamycin-2-phosphate, Sigma) 30 mg/kg body weight. After 16 hours, they were dosed i.g. with spores or vegetative cells of wild-type or mutant strains. Animals were monitored for symptoms of disease progression and culled upon reaching the clinical endpoint. The symptoms of CDI were scored as severe/clinical endpoint (wet tail >2 cm, high lethargy), mild (wet tail <2 cm), or healthy. Feces were sampled daily and ceca taken from culled animals.

#### Mice

The 50% infectious dose (ID_50_) was determined in mice (6–8 weeks old, female, Harlan UK Ltd) as described previously [17]. Animals were dosed i.g. with clindamycin (30 mg/kg) and challenged with spores (10^1^, 10^2^, and 10^3^ CFU) of strain 630 or *cotE* mutants. Ceca were removed 24 hours post-infection and colonization was defined by the presence of toxins and spore CFU counts 2 logs higher than the initial dose.

### Statistical Analysis

Statistical analysis was calculated and significance determined (*P* < .05) using the Welch *t* test for unequal variance. All statistical analyses were performed using GraphPad Prism software.

## RESULTS

### Generation of CotE Mutants and Their Complements

Two mutants of *cotE* and their complements have been described previously [12]. *cotE::CT220s* carries an insertion (*cotE::CT220s*) at the N-terminus preceding both the peroxiredoxin and chitinase domains, while the insertion in *cotE::CT1203s* is centrally positioned and precedes the C-terminal chitinase domain (Supplementary Figure 1*A*). For simplicity, these mutants will be described here as CotE^–^ (*cotE::CT220s*) and CotEC^–^ (*cotE::CT1203s*). To demonstrate the integrity of both mutants, we showed that each carried 2 copies of the *erm* gene, whereas the isogenic 630Δerm parent strain carried 1 copy (Supplementary Figure 1*B*). This confirmed the presence of 1 ClosTron insertion on the chromosome, which we further verified using DNA sequencing. Therefore, the phenotypes we observe for the 2 *cotE* mutants can be directly assigned to the absence of CotE or of course due to a pleiotropic effect (eg, disruption or missassembly of the spore coat resulting from the absence of an intact CotE). Using polyclonal antisera raised in mice to the N-terminal (residues 1–234) and C-terminal (residues 381–712) domains of CotE, we confirmed that neither mutant produced CotE (Supplementary Figure 1*C*). In previous work, we have reported a pRPF185-CotE complementation plasmid that carried full-length *cotE* [12]. The *cotE* mutant cells carrying this plasmid produced spores that expressed CotE (Supplementary Figure 1*D*). Functional complementation of the CotE mutant phenotype was demonstrated in this work and is described in more detail below.

### CotE Enables Binding of Spores to Mucus

**Figure 1.**
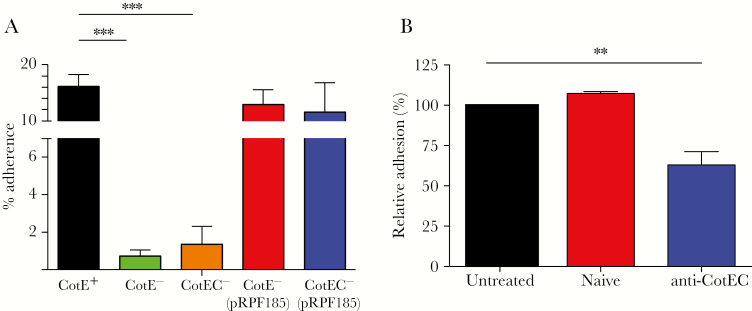
CotE is essential for spore binding to mucus-producing HT29-MTX cells. *A*, Spores of wild-type (630Δerm, *cotE*^+^), *cotE*^*–*^, and *cotEC*^*–*^ isogenic strains or *cotE* mutants carrying the pRPF185-CotE plasmid were added to mucus-producing HT29-MTX monolayers (multiplicity of infection of 100:1) aged 14 days. Non-adherent spores were removed by washing and the adherent spores were determined by plating on brain heart infusion broth supplemented with yeast extract, cysteine and sodium taurocholate agar. The experiment was independently repeated 3-times. Error bars represent standard deviations. **P ≤ .01, ***P ≤ .001 by the Welch unequal variance *t* test. *B*, Spores of 630Δerm (CotE^+^) were preincubated with anti-CotEC antibody or naive serum before adding to HT29-MTX cells. Non-adherent spores were removed by washing, and adherent spores were determined by plating on BHISS agar. Data for untreated spores and spore incubated with naive of anti-CotEC serum are shown. The experiment was independently repeated 3 times. Error bars represent standard deviations.

Adhesion of mutant and wild-type spores to mucus was evaluated using the HT29-MTX human cell line that secretes MUC2 mucins typically found in the small and large intestine and therefore more physiologically relevant for studies of pathogen–mucus interactions [18]. Wild-type 630Δerm spores exhibited higher levels of adhesion (15%–20%) to HT29-MTX cells compared to both *cotE* mutants, which showed significantly (*P* < .001) lower levels of adhesion (Figure 1A). In *cotE* mutant strains carrying the pRPF185-CotE plasmid, levels of adhesion were equivalent to wild type demonstrating functional, in trans, complementation of the CotE phenotype. Image analysis of antibody-labeled spores adhering to HT29-MTX cell lines (day 14) was also used to confirm that wild-type spores were more abundant on mucus-producing HT29-MTX monolayers (Supplementary Figure 2). In a second, ex vivo, approach we measured adhesion of wild-type and mutant spores to ileum and cecum biopsies obtained from a piglet (Supplementary Figure 3). For both *cotE* mutants, spore adhesion to ileum and cecum explants was significantly reduced compared with adhesion of wild-type spores at both incubation times. A further experiment, confirming that *cotE* is involved in binding of spores to mucus, was performed using wild-type spores preincubated with antibodies recognizing the C-terminal domain of *cotE* before being added to HT29-MTX cells. The data showed that adhesion of wild-type spores could be significantly reduced by preadsorption with CotE antibodies (Figure 1B).

### CotE Mediates Spore Binding to Mucin and Enhances Colonization

**Figure 2.**
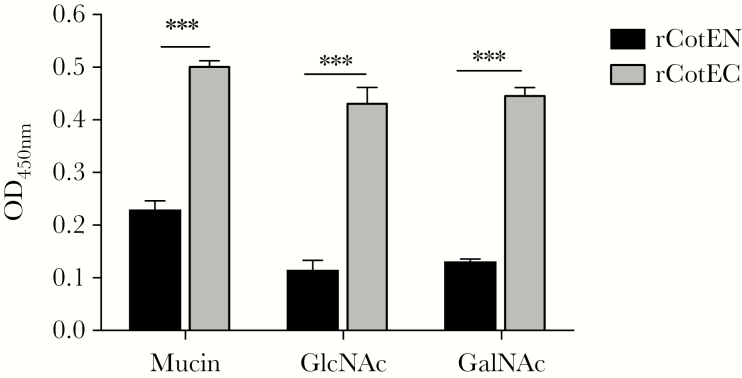
CotE binds to mucin via GlcNAc and GalNAc. The binding of proteins corresponding to the N-terminal (rCotEN) and C-terminal (rCotEC) domains of CotE to mucin or ligands was measured by enzyme-linked immunosorbent assay (ELISA). Mucin or ligands were first coated onto ELISA plates and either rCotEN or rCotEC then applied. Bound protein, after washing, was measured by ELISA. The experiment was independently repeated 3 times. Error bars represent standard deviations. ***P ≤ .001 by the Welch unequal variance *t* test. Abbreviation: OD_450_, optical density 450 nm.

To dissect CotE’s affinity for mucus, we used an ELISA-based method to examine binding of recombinant CotE protein fragments to mucin, the main component of mucus, as well as GlcNAc and GalNAc. GlcNAc is the repeating unit in chitin and is also present in mucin glycoproteins [19] and glycosylated proteins present on intestinal epithelial cells [20]. GalNAc is the C4 epimer of GlcNAc and is found (together with GlcNAc) in mucin. We observed significantly more binding of rCotEC, comprising the C-terminal chitinase domain, to mucin, GlcNAc, and GalNAc compared to rCotEN that carried the N-terminal peroxiredoxin domain (Figure 2).

In many pathogenic bacteria, binding to mucus and mucin is a mechanism for attachment to the intestinal epithelium and host colonization [19, 21]. We hypothesize that CotE enables *C. difficile* spores to accomplish the same. To address this, we determined the ID_50_ of mutants in a murine model (Table 1) as previously described by Phetcharaburanin et al [17]. In this model, animals do not show symptoms of disease, and colonization is measured by the presence of toxin and CFU (2–3 logs higher CFU than initial dose) in the cecum. Wild-type 630 spores had an ID_50_ of 10^1^ whereas values for the *cotE*^*–*^ (10^3^) and *cotEC*^*–*^ (10^2.3^) mutants were 1–2 logs higher, demonstrating that CotE had a role in the ability of spores to infect and colonize the host.

**Table 1.** The 50% Infectious Dose of *Clostridium difficile* Strain 630 and Mutant Spores in a Murine Model^a^

Strain	50% Infectious Dose
CotE^+^	10^1^
CotE^–^	10^3^
CotEC^–^	10^2.3^

^a^Mice are considered positive for *Clostridium difficile* infection when cecum tests positive for toxin and colony-forming units 2 logs higher than initial dose.

### CotE Facilitates Degradation of Mucin

**Figure 3.**
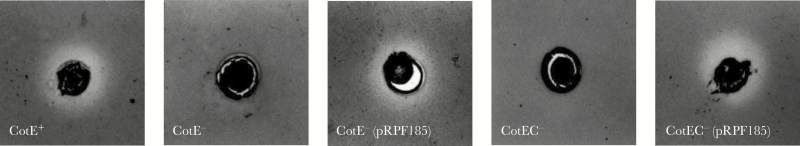
CotE promotes degradation of mucin. Mucin degradation assays for wild-type (630Δerm), *cotE* mutants, and complemented strains (carrying pRPF185-CotE). Spores were applied to wells cut in agarose containing mucin and were incubated aerobically for 48 h at 37°C before staining with amido black.

Chitin-binding proteins in bacteria can also function as mucolytic enzymes and therefore we investigated CotE for this trait [22, 23]. Using a modified mucin degradation assay [24], we showed mucin degradation mediated by CotE. After 48 hours of incubation, a zone or halo of mucin degradation was observed in amido black–stained mucin agarose wells containing wild-type spores (Figure 3). By contrast, very limited degradation was apparent in wells containing an identical number of spores for either of the *cotE* mutants. This phenotype, however, was restored in *cotE* mutant spores carrying pRPF185-CotE. Vegetative cells of *C. difficile* have been shown previously to be unable to degrade mucin [25], and we have confirmed this also with our wild-type and mutant strains (data not shown). Degradation of mucin by CotE was confirmed using fluorescently labeled mucin and reverse-phase high-performance liquid chromatography analysis (Supplementary Figure 4).

### CotE Enhances Virulence of CDI in Hamsters

**Figure 4.**
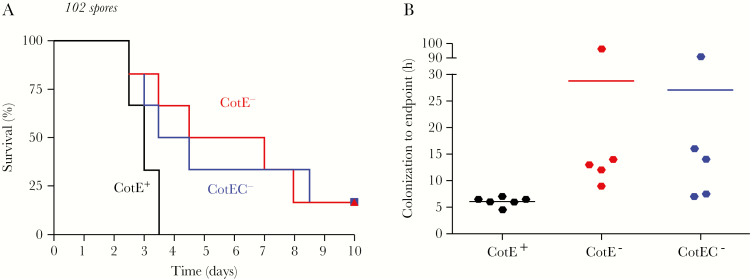
Absence of CotE slows progression of *Clostridium difficile* infection. Groups of hamsters (n = 6) were administered clindamycin and, 16 h later, dosed by intragastric gavage with spores (10^2^) of *C. difficile* 630 (wild-type, *cotE*^*+*^), *cotE*^*–*^, and *cotEC*^*–*^. *A*, Kaplan–Meier survival. *B*, Time from initial symptoms (wet tail and/or presence of fecal toxins) to clinical endpoint (virulent phase) are shown. Note that 1 hamster from each mutant group was not colonized and did not show symptoms.

**Figure 5.**
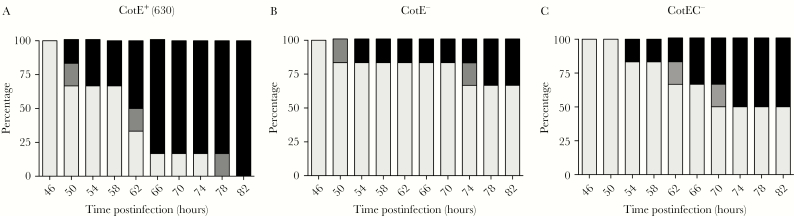
Reduced symptoms with the *cotE* mutants. Symptoms in the hamster study of Figure 4 displayed according to severity. *A*, Animals dosed with wild-type 630 spores. *B*, CotE^–^ spores. *C*, CotEC^–^ spores.

Degradation of mucin by intestinal pathogenic bacteria has been shown to contribute to virulence [19]. Accordingly, we investigated the effect of CotE on virulence using the hamster model of infection [26]. In this model, the colonization of the host can be observed from specific symptoms, or the presence of toxins and/or spore CFU in the cecum or feces. Virulence is reflected by survival rate and/or by the time from colonization to clinical endpoint (severe symptoms). In both mutants, 17% of hamsters were not colonized and survived until the end of the experiment (10 days) whereas with wild type, 100% of hamsters were colonized and eventually succumbed to infection (Figure 4A). While the time from colonization to clinical endpoint for 630 was approximately 6 hours, with CotE^–^ and CotEC^–^ the time was 28 and 27 hours, respectively (Figure 4B). Interestingly, among colonized hamsters (5/6), there was 1 hamster from each of the mutant groups which was positive for toxin in feces but developed no symptoms and only reached clinical endpoint after approximately 90 hours (Figure 4B). At 82 hours post-inoculation, 100% of hamsters infected with strain 630 spores had reached clinical endpoint, whereas with *cotE*^*–*^ and *cotEC*^*–*^ only 34% and 50%, respectively, had become moribund (Figure 5A–C). Once hamsters reached clinical endpoint, ceca were checked to confirm the presence of toxins and *C. difficile* spores at roughly equivalent levels between groups (Supplementary Figure 5). To rule out the possibility that the delay in symptoms and morbidity of hamsters could be due to a difference in toxin production, we checked the kinetics of toxin production both in vitro and in vivo and confirmed that they were of essentially equivalent levels between all 3 strains (Supplementary Figure 6). The germination rate of the mutant and wild-type spores has also been checked and confirmed to be the same (Supplementary Figure 7). The reduced virulence of *cotE* mutant spores observed here in hamsters has been independently repeated (Supplementary Figure 8).

**Figure 6.**
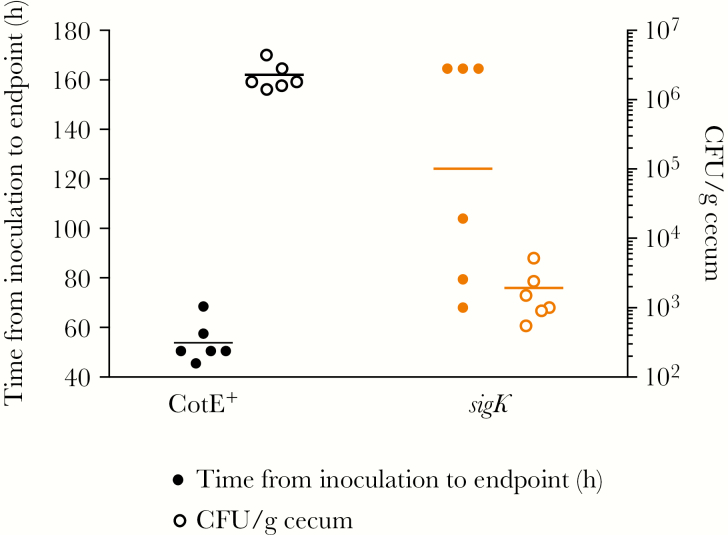
*sigK* mutants display lower virulence in hamsters. Groups of hamsters were administered clindamycin and then, 16 h later, dosed by intragastric gavage with vegetative cells (10^6^) of *Clostridium difficile* 630 (wild-type, *cotE*^*+*^) or JP051 (*sigK*^*–*^). Data represented as time from inoculation of *C. difficile* vegetative cells until time of clinical endpoint combined with heat-resistant spore counts from cecum. Abbreviation: CFU, colony-forming units.

To investigate whether sporulation affects virulence, we used vegetative cells of wild type and a sporulation defective *sigK*^*–*^ mutant to infect hamsters following the same dosing regime as that used above with spores. Mutation of *sigK* (encoding a sporulation-specific sigma factor controlling spore coat biosynthesis [27]) will affect the terminal stages of sporulation-specific gene expression in the mother cell chamber and, unlike *spoOA*, is less likely to exert pleiotropic effects on expression of additional virulence genes. While it took 72 hours for all strain 630–infected hamsters to reach clinical endpoint, 168 hours was required for all *sigK*^*–*^ infected animals to succumb (Figure 6). Since hamsters dosed with *sigK*^*–*^ cells exhibited delayed mortality, we ruled out the possibility of differential toxin production in vivo by measuring toxin kinetics in mice (Supplementary Figure 9). Note that in cecum samples, a low spore CFU count was found as the *sigK*^*–*^ mutant being oligosporogenous is presumably able to produce a very low level (approximately 4 logs less than 630 per gram of feces) of functional spores (Figure 6) [28]. These results suggest that (*i*) spores contribute to the virulence of CDI, and (*ii*) CotE, and potentially additional spore-associated proteins, are involved in colonization.

## DISCUSSION

Our study provides important insights into the role of the spore in CDI: first, that the spore is responsible for initial colonization by targeting and binding mucus enabling the establishment of infection and, second, that the spore is an active agent in the acute stage of infection. Although an interaction of the spore with mucus might not be unexpected, we can now attribute this to at least 1 spore-associated protein, CotE, a bifunctional peroxiredoxin/chitinase that is embedded in the outermost layers of the spore. What is surprising is that this spore enzyme is functionally active and able to bind directly to mucin monomers (the glycoproteins GlcNAc and GalNAc) and directly or indirectly facilitate mucin degradation. Some chitinases are thought to degrade mucus [22], and chitin binding proteins are considered colonization factors that have been found in several other important pathogens including *Listeria monocytogenes* [29], *Legionella pneumophila* [30], and *Escherichia coli* [31]. In each case, the proteins are secreted and have a direct role in virulence, colonization, and infection. *Vibrio cholerae* is a well-studied example where 2 proteins, GbpA and ChiA2 (1 of 2 chitinases in *V. cholerae*), act synergistically to bind and degrade both chitin and mucin. GbpA is a chitin-binding protein that is able to recognize GlcNAc present in chitin as well as on the surface of epithelial cells, enabling colonization of host cells by *V. cholerae* [21].

The adhesion of intestinal pathogenic bacteria to the gastrointestinal tract is a crucial aspect of host colonization as it prevents them being mechanically cleared [32]. In the case of *C. difficile,* the spore is the transmissible entity and here we show that for the successful establishment of infection, the spore must also adhere to the host. Interestingly, Theriot et al demonstrated that, within the small intestine, spores are able to germinate regardless of antibiotic perturbation [33]. It is therefore possible that spores are able to initiate colonization in the small bowel in the absence of microbiota disruption and that an intact microbiota is required to suppress vegetative *C. difficile* outgrowth to prevent clinical disease. Hong et al recently found that mucosal antibodies against the C-terminal of toxin A also recognize the spore and vegetative cells of *C. difficile* and prevents CDI in hamsters by blocking colonization [15]. We demonstrate that the spore binds to the gastrointestinal tract prior to germination to initiate CDI via interactions mediated by CotE. In our study, animals (both mice and hamsters) infected with spores lacking CotE show a marked reduction in colonization. It is important to consider that several adhesins may be responsible for initial spore attachment. In support of this, Phetcharaburanin et al identified hair-like appendages comprised of the BclA1 protein that make “first contact” with the host mucosa [17]. However, this study saw no differences between the severity of CDI caused by mutant BclA1 and wild-type 630 in hamsters.

In our study, spores appear able to enhance the severity of disease once initial colonization has been established. This is clear from hamster studies showing that the time required to develop symptoms in colonized animals was markedly delayed in animals dosed with spores devoid of CotE. To explain this, we can envisage several scenarios. One possible explanation could be that by degrading mucus, CotE facilitates transit of toxins to the underlying epithelial cells and so mucin degradation is an integral step in pathogenesis. Another possibility is that the severity of disease is due to the combined action of toxins *and* spores interacting with the large number of macrophages and neutrophils produced during the acute stages of infection leading to the production of inflammatory cytokines [3]. In support of this we have shown, in vitro, that spores are inflammatory and that this response is significantly reduced in the absence of CotE (Supplementary Figure 10). The spore acting as a virulence factor has been shown in *Bacillus anthracis* where a spore surface superoxide dismutase enhances virulence by as much as 40-fold in a mouse model of anthrax [34].

Over the past decade, a multitude of studies have been conducted to understand the severity of epidemic and hypervirulent 027/B1/NAP1 strains, but precise causative mechanisms have yet to be found and links between high sporulation rates and hypervirulence remain inconclusive [35, 36]. A direct role for the spore in acute infection has implications for the concept of hypervirulence, a phenomenon that so far lacks clarity. Most compelling are recent in vivo studies showing (*i*) increased persistence of hypervirulent 027 strains (R20291) in the gastrointestinal tract [17] and (*ii*) increased levels of intestinal sporulation that correlated with increased severity of disease [9]. Using isogenic strains, we show that administration of cells of a *sigK* mutant that exhibits very low levels of spores results in an extended period of infection, post-colonization. One straightforward explanation is that increased spore numbers together with greater intestinal persistence exacerbate symptoms of CDI by CotE-mediated mucin degradation and/or inflammation.

## Supplementary Data

Supplementary materials are available at *The Journal of Infectious Diseases* online. Consisting of data provided by the authors to benefit the reader, the posted materials are not copyedited and are the sole responsibility of the authors, so questions or comments should be addressed to the corresponding author.

## Supplementary Material

Supplementary Material

Supplementary Figures 1-10

## Notes

***Acknowledgments.*** We thank Dr Ian Teo (Imperial College, United Kingdom) for advice on the quantitative reverse-transcription polymerase chain reaction assays.

***Financial support.*** This work was supported by the Medical Research Council (grant number MR/K015354/1 to S. M. C.) and EU Era-Net (grant number ANIHWA 031A357 to S. M. C. and J. Z.).

***Potential conflicts of interest.*** All authors: No reported conflicts of interest. All authors have submitted the ICMJE Form for Disclosure of Potential Conflicts of Interest. Conflicts that the editors consider relevant to the content of the manuscript have been disclosed.
