# Supplementary material for: The Spore Coat Protein CotE Facilitates Host Colonization by Clostridium difficile
Source: J Infect Dis. 2017 Sep 15;216(11):1452–9. doi: 10.1093/infdis/jix488 (PMC5853579; doi:10.1093/infdis/jix488)
Supplement: Supplementary Material [file jix488_suppl_supplementary_material.docx]

**Supplementary Methods**

**Growth of *C. difficile* and preparation of spores**

Vegetative *C. difficile* was grown in TGY medium overnight, washed once in PBS and the pellet resuspended in an equal volume of PBS giving a final concentration of 1 X 10^7^ cells/ml [1]. Spores of *C. difficile* were prepared by growth on SMC agar plates using an anaerobic incubator (Don Whitley, UK) as described previously [2]. After growth for seven days at 37^o^C spores were harvested and the spore pellet further purified using centrifugation through a 20% to 50% Histodenz gradient (Sigma) as described elsewhere [3]. Spore CFU was determined by heat treatment (60°C, 20 min.) and plating on BHISS agar plates (Brain heart infusion agar containing 0.1% (w/v) L-cysteine, 5 mg/ml yeast extract and the spore germinant sodium taurocholate (0.1% w/v). Spores of the complemented strains were prepared in the same way, but all media, including agar plates was supplemented with 15μg/ml thiamphenicol and 500ng/ml anhydrous tetracycline (ATC).

**Extraction of spore coat proteins**

Spores (1 X 10^8^) and vegetative cells were heated at 80^o^C for 20 min and then pelleted by centrifugation and suspended in 40 μl of extraction butter (Bolt (Thermo) LDS Sample Buffer 10μl, Bolt Reducing Agent 4μl, in 26 μl of deionized water containing 0.5μM PMSF and 0.052μg of benzamide) and incubated for 10 min. at 95^o^C. The suspensions were then centrifuged (10,000g, 4^o^C, 5 min.) and supernatants kept on ice until SDS-PAGE analysis.

**Adhesion of mutant *C. difficile* spores to HT29 and HT29-MTX cells**

The method used for adhesion of spores to HT29-MTX cell lines was as described previously [4]. Briefly, cells were seeded at 4 x 10^4^ cells/well in 24-well plates and grown for 14 days at 37ºC, 7.5% CO_2._ Mucus production was checked using alcian blue staining. On the day of experimentation, cell monolayers were washed once with Dulbecco’s Phosphate-Buffered Saline (DPBS) (Life-technologies, UK) and incubated with *C. difficile* spores at a multiplicity of infection (MOI) of 100 for 2h at 37ºC. Wells were washed five-times with DPBS to remove non-adherent bacteria and then cells lysed with 1ml of 0.5% Triton X-100 in PBS for 10 min. and adherent *C. difficile* spores were plated on BHISS agar. Percentage adhesion was calculated using the formula % adhesion = (CFU count / Initial number of spores added) x 100.

**Southern Blotting (Supp. Fig.1B)**

Genomic DNA was isolated from cultures of 630Δerm, CotE^-^ and CotEC^-^ cells grown in BHIS using a phenol-chloroform extraction method [5]. The probe was prepared by amplifying a 500bp sequence found within the *C. difficile* *erm^R^* gene of the ClosTron plasmid, pMTL007C, used for gene constructions. Primers used were ERMF (CGAAATTGGAACAGGTAAAGG) and ERMR (GCTTCCAATATTTATCTGGAACATC). The amplification product was purified using QIAquick PCR purification kit (Qiagen) and labeled with Dig-High Prime following the manufacturers instructions. Genomic DNA was digested with HindIII and run on an agarose gel (0.8%). DNA was transferred onto a Hybond-N+ membrane (Amsersham) using 0.4M NaOH as transfer buffer. The probe was denatured (100^o^C for 10 min.) and added to Dig High prime hybridization buffer and the membrane was hybridized overnight at 42^o^c. The membrane was washed, blocked, detected and developed following the manufacturers instructions; DIG High Prime DNA labeling and Detection Kit I (Roche, Cat. No 11745832910).

**Reverse-phase HPLC (Supp. Fig. 4)**

A freshly made solution of porcine mucin was labeled fluorescently with Rhodamine B isothiocyanate (RBITC, mixture of isomers, Sigma-Aldrich, 83692) and purified by gravity-flow chromatography using 10DG Desalting Columns filled with Bio-Gel® P-6DG Gel (Bio-Rad, 7322010), equilibrated in PBS pH 6.4 buffer prior to the. Equal aliquots of mucin spiked with the fluorescently labeled RBITC-mucin in PBS (pH 6.4) were added to purified wild type and mutant *cotEC^-^* spores of *C. difficile*. Suspensions were incubated for 36h at 37^o^C under anaerobic conditions with continuous gentle agitation. Mixtures were pelleted (10,000g, 10 min.). SDS was the added to all samples (final concentration 0.2% w/v) to stop any further interactions and to ensure stability and solubility. Before HPLC analysis samples were again pelleted. A TSKgel® Size Exclusion HPLC Column (PW_XL_-Type, L. x I.D. 300mm x 7.8mm) was used to simultaneously exchange buffer in mucin samples and then to conduct reverse phase separation of the absorbed and washed mucins by gradient elution using mobile phases with decreasing salt concentration and increased concentration of organic modifier. A FLEXAR programmable HPLC setup included a quaternary solvent delivery system, autosampler and a column oven (30⁰C). The eluted mucins were detected using an Altus A-10 FL fluorescence detector (excitation was at 550 nm, emission was at 575 nm). All samples were injected in 50% PBS pH 6.4 (0.4 ml/min.) and the isocratic flow was maintained for up to 30 min. to allow elution of hydrophilic sample constituents. Under these conditions mucins remain associated with weakly hydrophobic hydroxylated polymethacrylate PW_XL_ polymer stationary phase, thus allowing an extended washing and equilibration cycle (column eluates were monitored by measuring fluorescence ex.550nm/em.575nm). For gradient elution the mobile phase was changed from 0/0/100 to 100/0/0 A/B/C in 25 min. (eluent A: 100% Methanol; eluent B: deionized water; eluent C: 50% PBS, pH 6.4). The column was regenerated and re-equilibrated after each separation by washing with individual eluents A, B and C (15 min. each wash).

**Cytotoxicity assays**

Assays followed that described previously [6]. Overnight cultures of *C. difficile* in TY broth were used to inoculate a starting culture. Samples were taken at 6, 12, 24 and 48h, centrifuged (10,000g, 10 min.) and supernatants filter-sterilized through 0.2μ filters. The samples were then normalised proportionally according to the OD taken at collection using PBS. Samples were serially diluted in PBS and 10 μl was added to 90μl of 2% FCS DMEM media above confluent HT29 or VERO cells giving a 1:10 dilution. Cells were incubated for 24h at 37^o^C, 5% CO_2_ for 24h before being scored for toxin endpoints using an Olympus CKX41. End point titers were taken at the point where cell rounding was less than 50%.

1. Paredes-Sabja D, Bond C, Carman RJ, Setlow P, Sarker MR. Germination of spores of *Clostridium difficile* strains, including isolates from a hospital outbreak of *Clostridium difficile*-associated disease (CDAD). Microbiology **2008**; 154:2241-50.

2. Permpoonpattana P, Hong HA, Phetcharaburanin J, et al. Immunization with *Bacillus* spores expressing toxin A peptide repeats protects against infection with *Clostridium difficile* strains producing toxins A and B. Infection and immunity **2011**; 79:2295-302.

3. Phetcharaburanin J, Hong HA, Colenutt C, et al. The spore-associated protein BclA1 affects the susceptibility of animals to colonization and infection by *Clostridium difficile*. Mol Microbiol **2014**; 92:1025-38.

4. Hong HA, Hitri K, Hosseini S, et al. Mucosal Antibodies to the C-terminus of Toxin A Prevent Colonization of *Clostridium difficile* Infection and Immunity **2017**; 85.

5. Wren BW, Tabaqchali S. Restriction endonuclease DNA analysis of *Clostridium difficile*. J Clin Microbiol **1987**; 25:2402-4.

6. Kuehne SA, Cartman ST, Heap JT, Kelly ML, Cockayne A, Minton NP. The role of toxin A and toxin B in *Clostridium difficile* infection. Nature **2010**; 467:711-3.
