# Supplementary material for: The Spore Coat Protein CotE Facilitates Host Colonization by Clostridium difficile
Source: J Infect Dis. 2017 Sep 15;216(11):1452–9. doi: 10.1093/infdis/jix488 (PMC5853579; doi:10.1093/infdis/jix488)
Supplement: Supplementary Figures 1-10 [file jix488_suppl_supplementary_figure_1-10.pptx]

## Slide 1
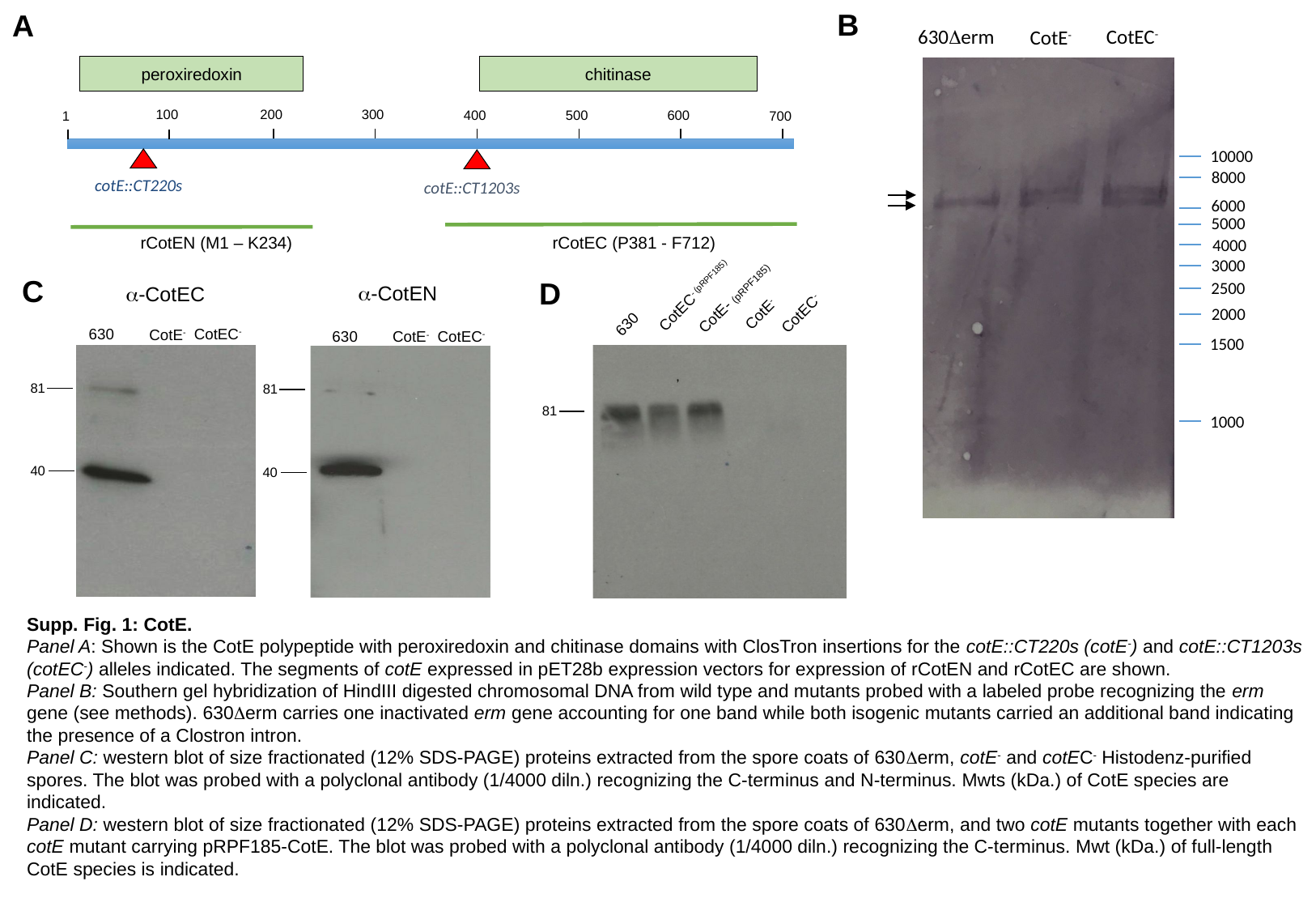

B
A
630Derm
CotEC-
CotE-
peroxiredoxin
chitinase
100
200
300
400
500
600
1
700
cotE::CT220s
cotE::CT1203s
rCotEN (M1 – K234)
rCotEC (P381 - F712)
10000
8000
6000
5000
4000
3000
C
D
2500
a-CotEN
a-CotEC
CotEC- (pRPF185)
 CotE- (pRPF185)
2000
CotEC-
 CotE-
630
CotEC-
630
 CotE-
CotEC-
630
 CotE-
1500
81
81
81
1000
40
40
Supp. Fig. 1: CotE.
Panel A: Shown is the CotE polypeptide with peroxiredoxin and chitinase domains with ClosTron insertions for the cotE::CT220s (cotE-) and cotE::CT1203s (cotEC-) alleles indicated. The segments of cotE expressed in pET28b expression vectors for expression of rCotEN and rCotEC are shown.
Panel B: Southern gel hybridization of HindIII digested chromosomal DNA from wild type and mutants probed with a labeled probe recognizing the erm gene (see methods). 630Derm carries one inactivated erm gene accounting for one band while both isogenic mutants carried an additional band indicating the presence of a Clostron intron.
Panel C: western blot of size fractionated (12% SDS-PAGE) proteins extracted from the spore coats of 630Derm, cotE- and cotEC- Histodenz-purified spores. The blot was probed with a polyclonal antibody (1/4000 diln.) recognizing the C-terminus and N-terminus. Mwts (kDa.) of CotE species are indicated.
Panel D: western blot of size fractionated (12% SDS-PAGE) proteins extracted from the spore coats of 630Derm, and two cotE mutants together with each cotE mutant carrying pRPF185-CotE. The blot was probed with a polyclonal antibody (1/4000 diln.) recognizing the C-terminus. Mwt (kDa.) of full-length CotE species is indicated.

## Slide 2
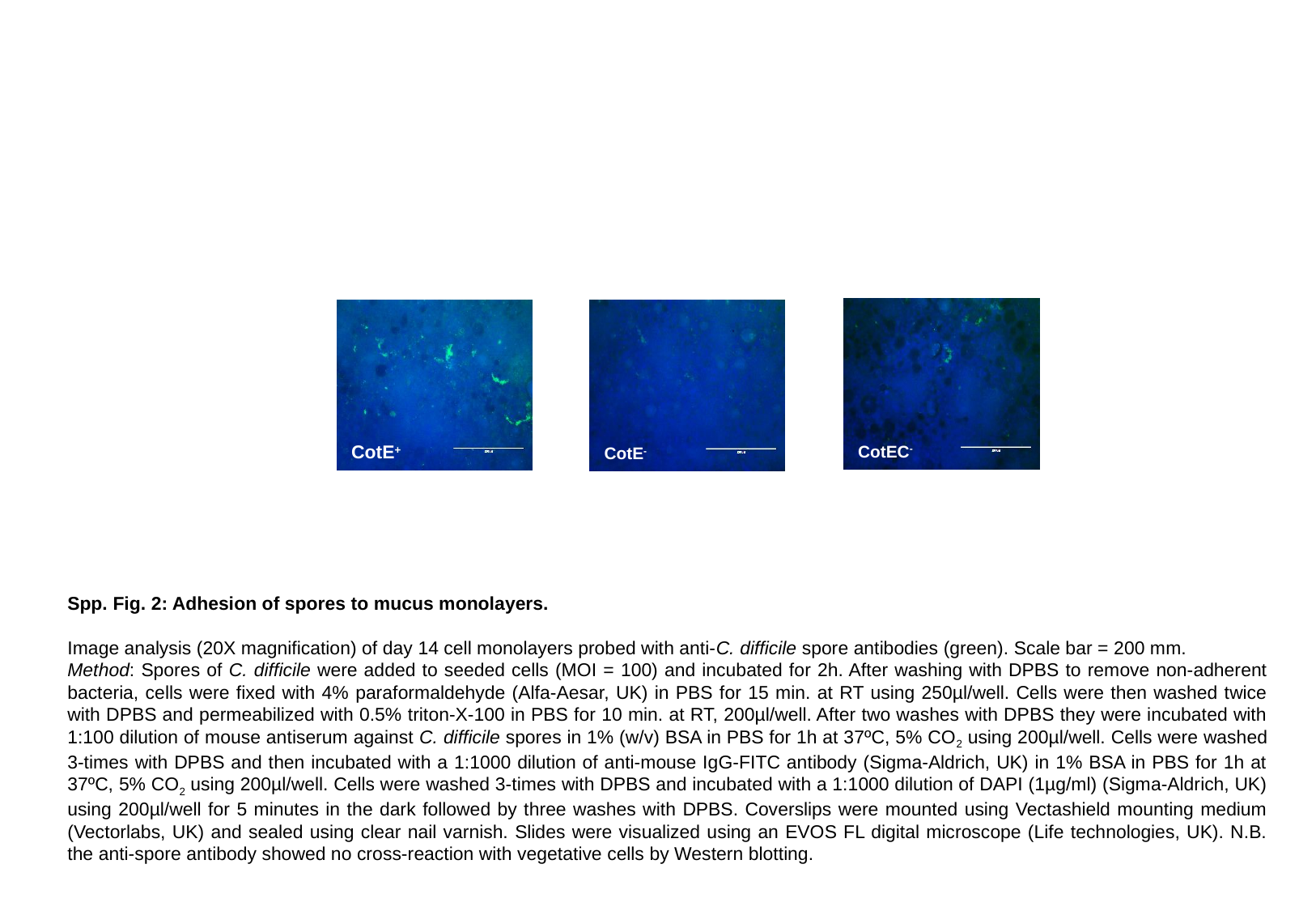

CotEC-
CotE+
CotE-
Spp. Fig. 2: Adhesion of spores to mucus monolayers.
Image analysis (20X magnification) of day 14 cell monolayers probed with anti-C. difficile spore antibodies (green). Scale bar = 200 mm.
Method: Spores of C. difficile were added to seeded cells (MOI = 100) and incubated for 2h. After washing with DPBS to remove non-adherent bacteria, cells were fixed with 4% paraformaldehyde (Alfa-Aesar, UK) in PBS for 15 min. at RT using 250µl/well. Cells were then washed twice with DPBS and permeabilized with 0.5% triton-X-100 in PBS for 10 min. at RT, 200µl/well. After two washes with DPBS they were incubated with 1:100 dilution of mouse antiserum against C. difficile spores in 1% (w/v) BSA in PBS for 1h at 37ºC, 5% CO2 using 200µl/well. Cells were washed 3-times with DPBS and then incubated with a 1:1000 dilution of anti-mouse IgG-FITC antibody (Sigma-Aldrich, UK) in 1% BSA in PBS for 1h at 37ºC, 5% CO2 using 200µl/well. Cells were washed 3-times with DPBS and incubated with a 1:1000 dilution of DAPI (1µg/ml) (Sigma-Aldrich, UK) using 200µl/well for 5 minutes in the dark followed by three washes with DPBS. Coverslips were mounted using Vectashield mounting medium (Vectorlabs, UK) and sealed using clear nail varnish. Slides were visualized using an EVOS FL digital microscope (Life technologies, UK). N.B. the anti-spore antibody showed no cross-reaction with vegetative cells by Western blotting.

## Slide 3
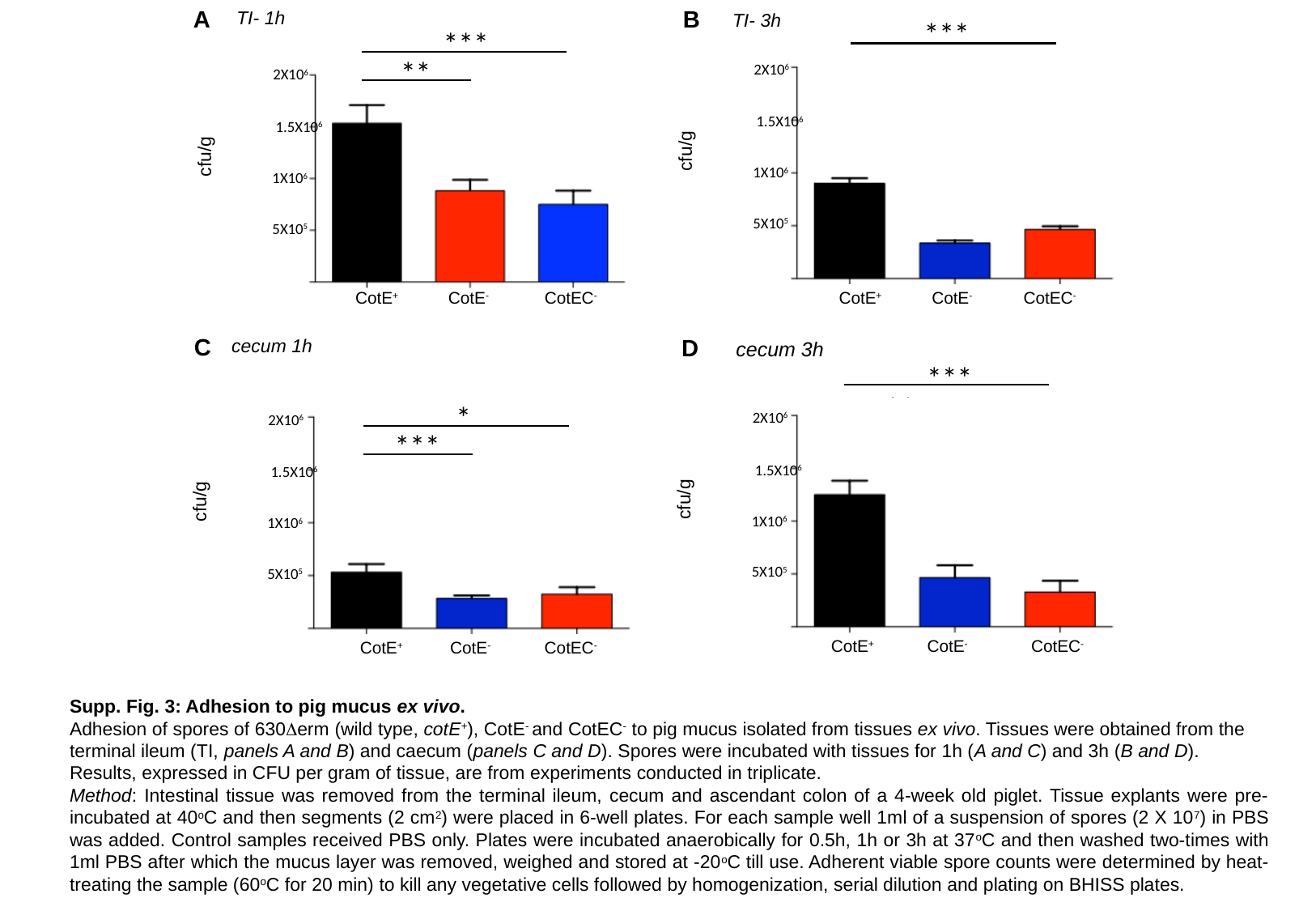

A
B
TI- 1h
TI- 3h
***
***
***
**
2X106
2X106
1.5X106
1.5X106
cfu/g
cfu/g
1X106
1X106
5X105
5X105
 CotE+ CotE- CotEC-
 CotE+ CotE- CotEC-
C
D
cecum 1h
cecum 3h
***
**
*
2X106
2X106
***
1.5X106
1.5X106
cfu/g
cfu/g
1X106
1X106
5X105
5X105
 CotE+ CotE- CotEC-
 CotE+ CotE- CotEC-
Supp. Fig. 3: Adhesion to pig mucus ex vivo.
Adhesion of spores of 630Derm (wild type, cotE+), CotE- and CotEC- to pig mucus isolated from tissues ex vivo. Tissues were obtained from the terminal ileum (TI, panels A and B) and caecum (panels C and D). Spores were incubated with tissues for 1h (A and C) and 3h (B and D). Results, expressed in CFU per gram of tissue, are from experiments conducted in triplicate.
Method: Intestinal tissue was removed from the terminal ileum, cecum and ascendant colon of a 4-week old piglet. Tissue explants were pre-incubated at 40oC and then segments (2 cm2) were placed in 6-well plates. For each sample well 1ml of a suspension of spores (2 X 107) in PBS was added. Control samples received PBS only. Plates were incubated anaerobically for 0.5h, 1h or 3h at 37oC and then washed two-times with 1ml PBS after which the mucus layer was removed, weighed and stored at -20oC till use. Adherent viable spore counts were determined by heat-treating the sample (60oC for 20 min) to kill any vegetative cells followed by homogenization, serial dilution and plating on BHISS plates.

## Slide 4
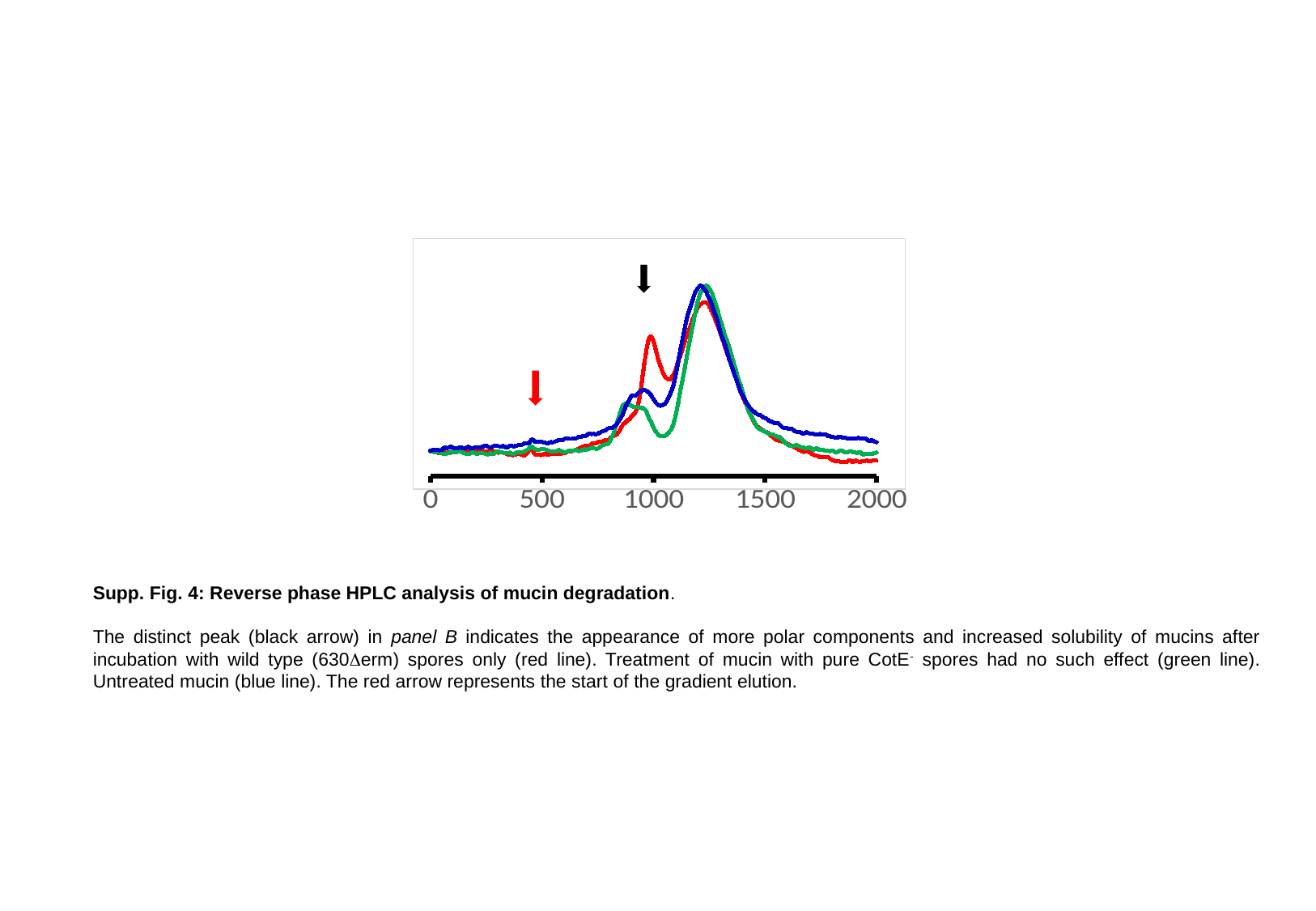

### Chart
| Category | No treatment | N | WT |
|---|---|---|---|
Supp. Fig. 4: Reverse phase HPLC analysis of mucin degradation.
The distinct peak (black arrow) in panel B indicates the appearance of more polar components and increased solubility of mucins after incubation with wild type (630Derm) spores only (red line). Treatment of mucin with pure CotE- spores had no such effect (green line). Untreated mucin (blue line). The red arrow represents the start of the gradient elution.

## Slide 5
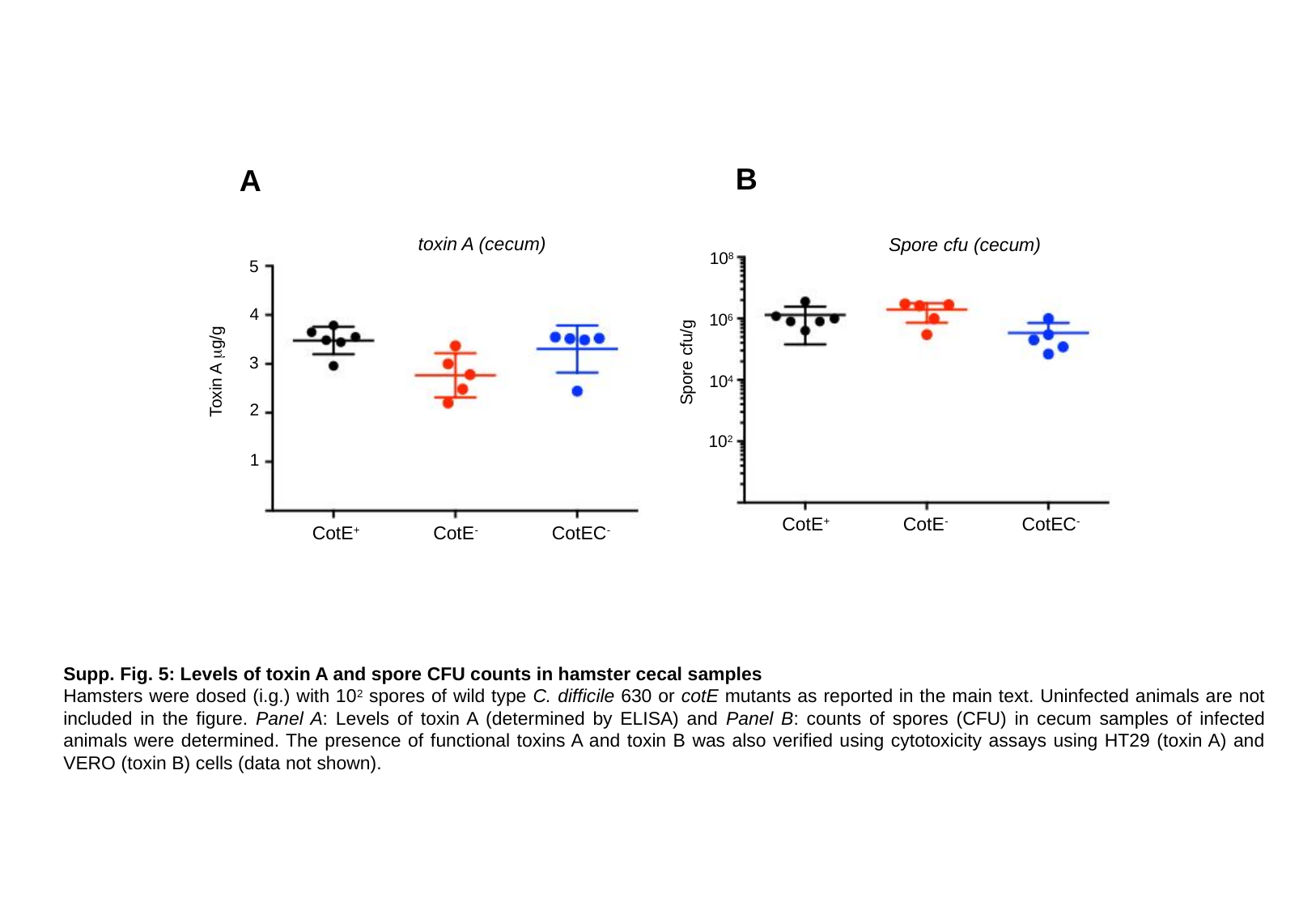

B
A
toxin A (cecum)
Spore cfu (cecum)
108
106
Spore cfu/g
104
102
5
4
3
Toxin A mg/g
2
1
 CotE+ CotE- CotEC-
 CotE+ CotE- CotEC-
Supp. Fig. 5: Levels of toxin A and spore CFU counts in hamster cecal samples
Hamsters were dosed (i.g.) with 102 spores of wild type C. difficile 630 or cotE mutants as reported in the main text. Uninfected animals are not included in the figure. Panel A: Levels of toxin A (determined by ELISA) and Panel B: counts of spores (CFU) in cecum samples of infected animals were determined. The presence of functional toxins A and toxin B was also verified using cytotoxicity assays using HT29 (toxin A) and VERO (toxin B) cells (data not shown).

## Slide 6
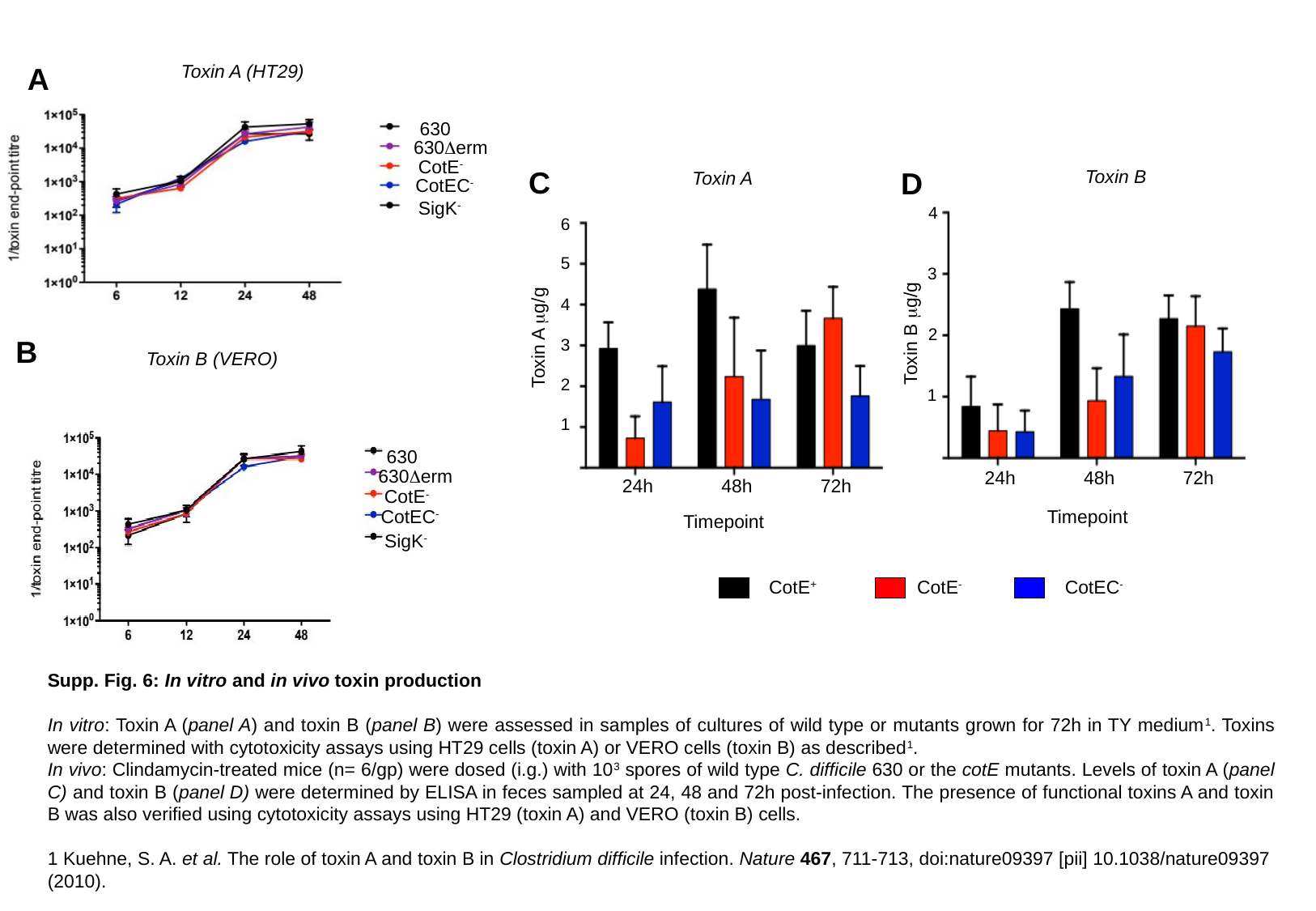

A
Toxin A (HT29)
630
630Derm
CotE-
C
D
Toxin B
Toxin A
CotEC-
SigK-
4
6
5
3
4
Toxin B mg/g
2
Toxin A mg/g
B
3
Toxin B (VERO)
2
1
1
630
630Derm
CotE-
CotEC-
SigK-
24h 48h 72h
24h 48h 72h
Timepoint
Timepoint
CotE+
CotE-
CotEC-
Supp. Fig. 6: In vitro and in vivo toxin production
In vitro: Toxin A (panel A) and toxin B (panel B) were assessed in samples of cultures of wild type or mutants grown for 72h in TY medium1. Toxins were determined with cytotoxicity assays using HT29 cells (toxin A) or VERO cells (toxin B) as described1.
In vivo: Clindamycin-treated mice (n= 6/gp) were dosed (i.g.) with 103 spores of wild type C. difficile 630 or the cotE mutants. Levels of toxin A (panel C) and toxin B (panel D) were determined by ELISA in feces sampled at 24, 48 and 72h post-infection. The presence of functional toxins A and toxin B was also verified using cytotoxicity assays using HT29 (toxin A) and VERO (toxin B) cells.
1 Kuehne, S. A. et al. The role of toxin A and toxin B in Clostridium difficile infection. Nature 467, 711-713, doi:nature09397 [pii] 10.1038/nature09397 (2010).

## Slide 7
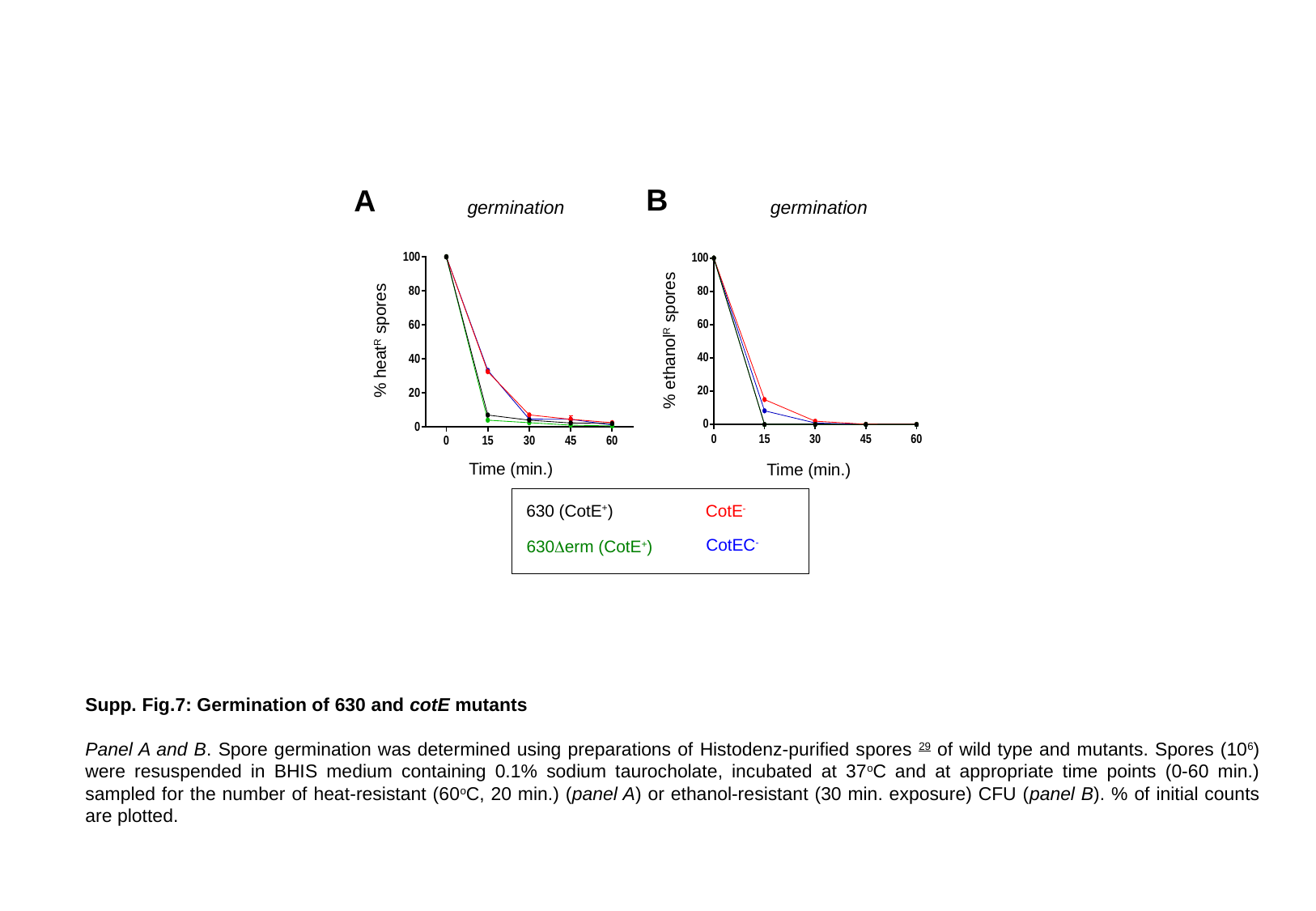

B
A
germination
germination
% ethanolR spores
% heatR spores
Time (min.)
Time (min.)
630 (CotE+)
CotE-
CotEC-
630Derm (CotE+)
Supp. Fig.7: Germination of 630 and cotE mutants
Panel A and B. Spore germination was determined using preparations of Histodenz-purified spores 29 of wild type and mutants. Spores (106) were resuspended in BHIS medium containing 0.1% sodium taurocholate, incubated at 37oC and at appropriate time points (0-60 min.) sampled for the number of heat-resistant (60oC, 20 min.) (panel A) or ethanol-resistant (30 min. exposure) CFU (panel B). % of initial counts are plotted.

## Slide 8
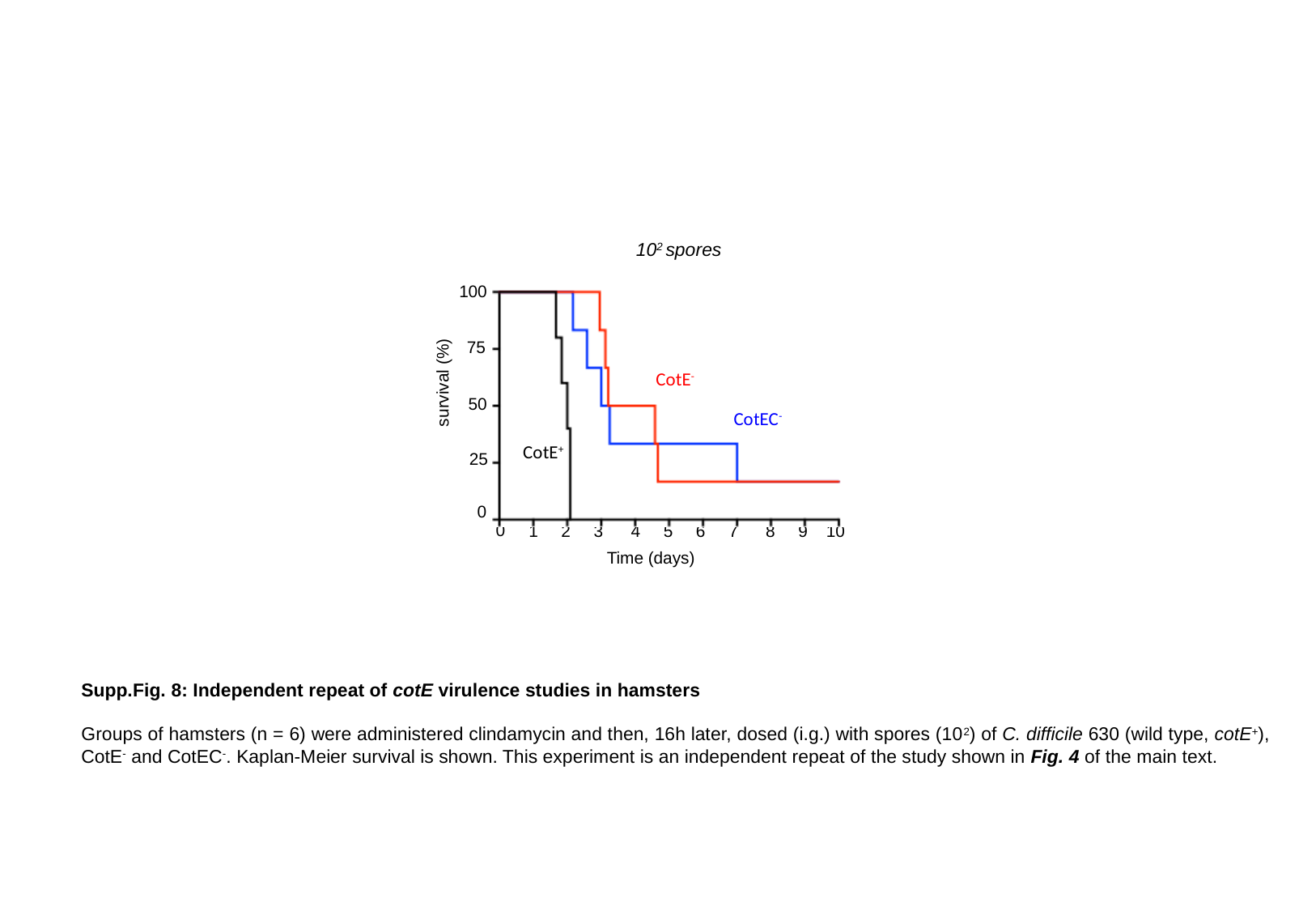

102 spores
100
75
CotE-
CotE-
survival (%)
50
CotEC-
CotE+
25
CotE+
CotEC-
0
0
 1 2 3 4 5 6 7 8 9 10
Time (days)
Supp.Fig. 8: Independent repeat of cotE virulence studies in hamsters
Groups of hamsters (n = 6) were administered clindamycin and then, 16h later, dosed (i.g.) with spores (102) of C. difficile 630 (wild type, cotE+), CotE- and CotEC-. Kaplan-Meier survival is shown. This experiment is an independent repeat of the study shown in Fig. 4 of the main text.

## Slide 9
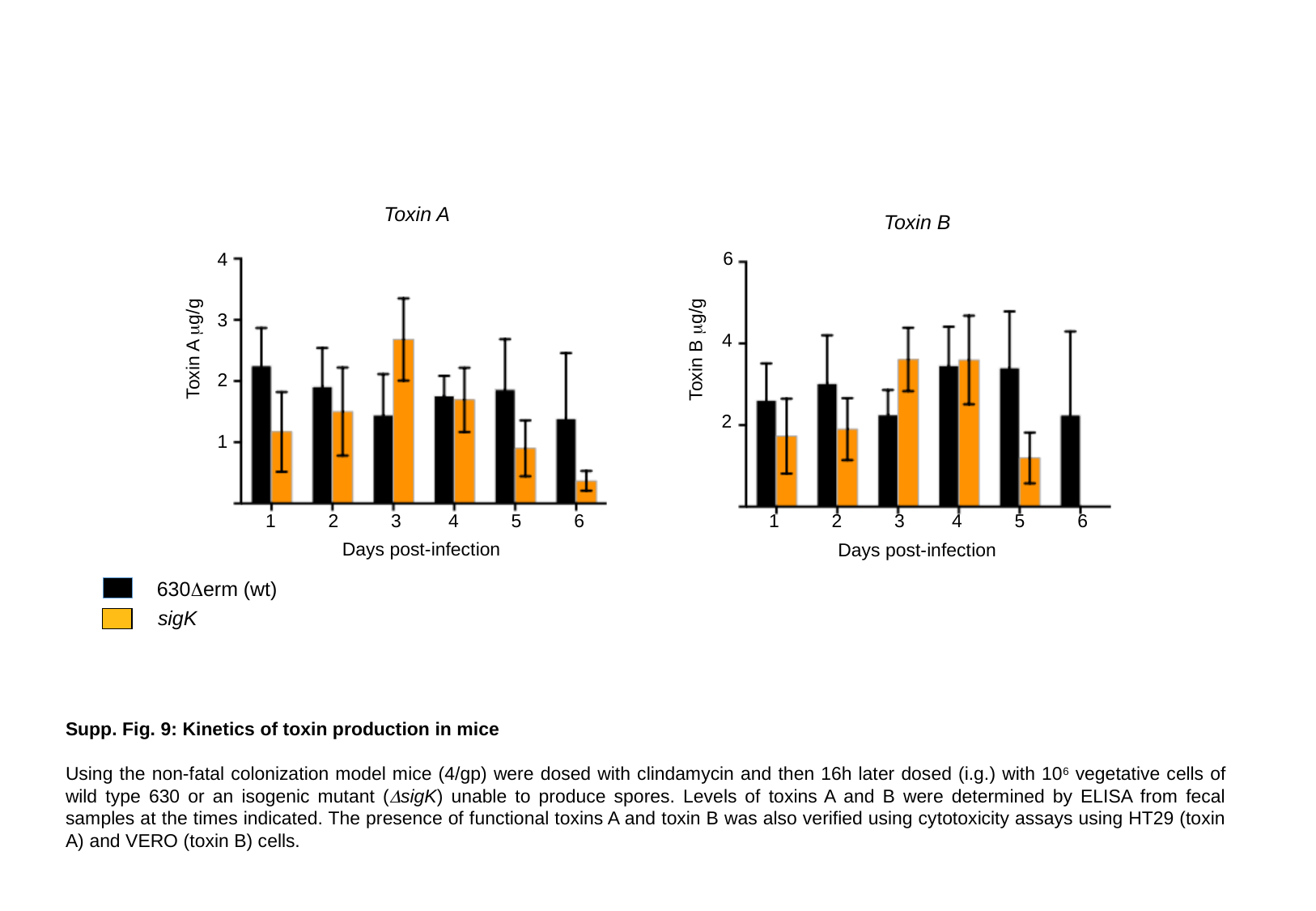

Toxin A
Toxin B
6
4
3
4
Toxin A mg/g
Toxin B mg/g
2
2
1
 1 2 3 4 5 6
 1 2 3 4 5 6
Days post-infection
Days post-infection
630Derm (wt)
sigK
Supp. Fig. 9: Kinetics of toxin production in mice
Using the non-fatal colonization model mice (4/gp) were dosed with clindamycin and then 16h later dosed (i.g.) with 106 vegetative cells of wild type 630 or an isogenic mutant (DsigK) unable to produce spores. Levels of toxins A and B were determined by ELISA from fecal samples at the times indicated. The presence of functional toxins A and toxin B was also verified using cytotoxicity assays using HT29 (toxin A) and VERO (toxin B) cells.

## Slide 10
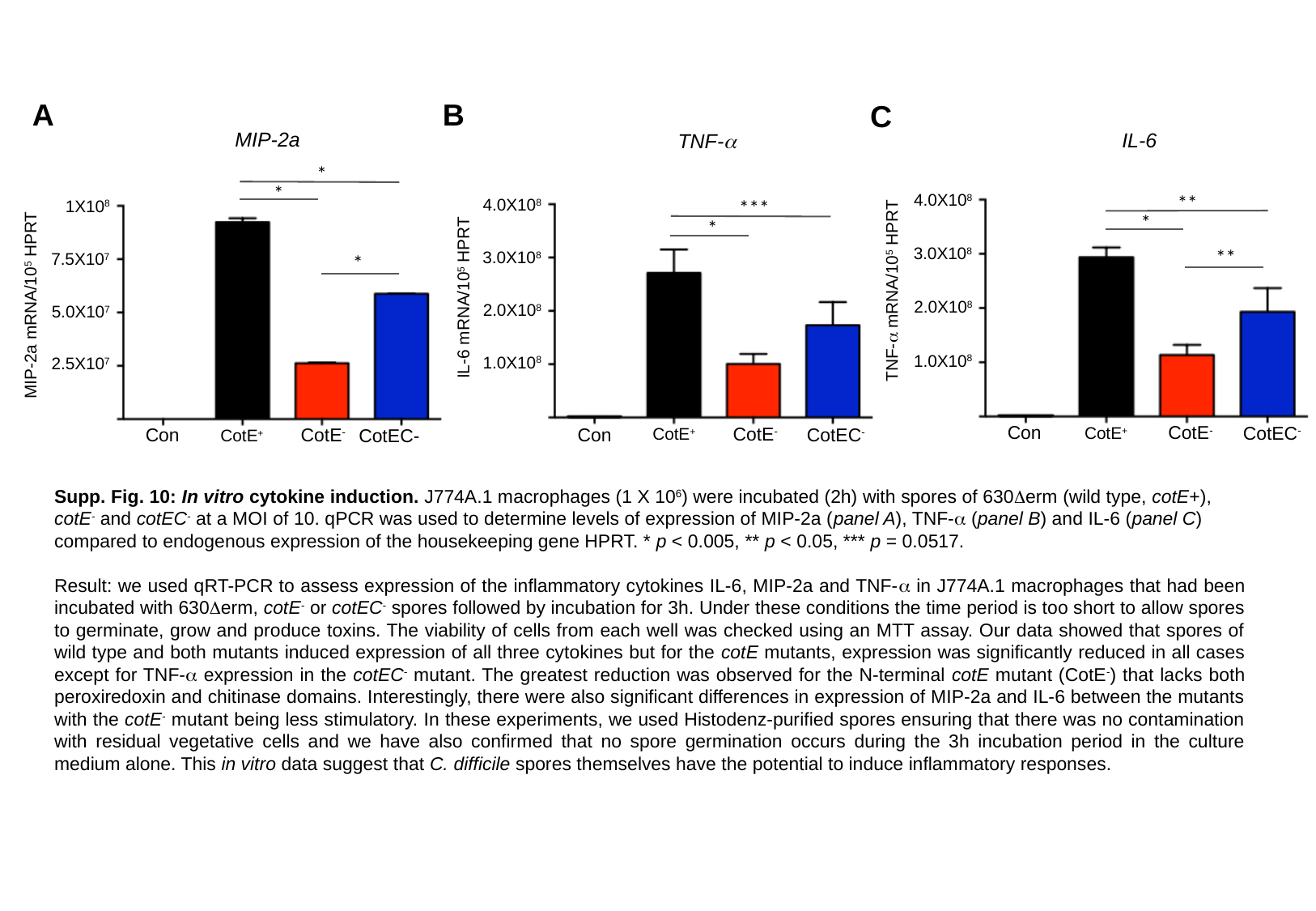

B
A
C
MIP-2a
IL-6
TNF-a
*
*
4.0X108
**
4.0X108
***
1X108
*
*
3.0X108
**
3.0X108
7.5X107
*
TNF-a mRNA/105 HPRT
IL-6 mRNA/105 HPRT
MIP-2a mRNA/105 HPRT
2.0X108
2.0X108
5.0X107
1.0X108
1.0X108
2.5X107
Con
CotE-
CotEC-
CotE+
CotE-
Con
CotE-
Con
CotEC-
CotE+
CotEC-
CotE+
Supp. Fig. 10: In vitro cytokine induction. J774A.1 macrophages (1 X 106) were incubated (2h) with spores of 630Derm (wild type, cotE+), cotE- and cotEC- at a MOI of 10. qPCR was used to determine levels of expression of MIP-2a (panel A), TNF-a (panel B) and IL-6 (panel C) compared to endogenous expression of the housekeeping gene HPRT. * p < 0.005, ** p < 0.05, *** p = 0.0517.
Result: we used qRT-PCR to assess expression of the inflammatory cytokines IL-6, MIP-2a and TNF-a in J774A.1 macrophages that had been incubated with 630Derm, cotE- or cotEC- spores followed by incubation for 3h. Under these conditions the time period is too short to allow spores to germinate, grow and produce toxins. The viability of cells from each well was checked using an MTT assay. Our data showed that spores of wild type and both mutants induced expression of all three cytokines but for the cotE mutants, expression was significantly reduced in all cases except for TNF-a expression in the cotEC- mutant. The greatest reduction was observed for the N-terminal cotE mutant (CotE-) that lacks both peroxiredoxin and chitinase domains. Interestingly, there were also significant differences in expression of MIP-2a and IL-6 between the mutants with the cotE- mutant being less stimulatory. In these experiments, we used Histodenz-purified spores ensuring that there was no contamination with residual vegetative cells and we have also confirmed that no spore germination occurs during the 3h incubation period in the culture medium alone. This in vitro data suggest that C. difficile spores themselves have the potential to induce inflammatory responses.
